# Alphapartitiviruses of *Heterobasidion* Wood Decay Fungi Affect Each Other's Transmission and Host Growth

**DOI:** 10.3389/fcimb.2019.00064

**Published:** 2019-03-26

**Authors:** Muhammad Kashif, Jaana Jurvansuu, Eeva J. Vainio, Jarkko Hantula

**Affiliations:** ^1^Forest Health and Biodiversity, Natural Resources Institute Finland, Helsinki, Finland; ^2^Department of Biology, University of Oulu, Oulu, Finland

**Keywords:** mycovirus, partitivirus, transmission, transcripts, *Heterobasidion annosum*, growth rate

## Abstract

*Heterobasidion* spp. root rot fungi are highly destructive forest pathogens of the northern boreal forests, and are known to host a diverse community of partitiviruses. The transmission of these mycoviruses occurs horizontally among host strains via mycelial anastomoses. We revealed using dual cultures that virus transmission rates are affected by pre-existing virus infections among two strains of *H. annosum*. The transmission efficacy of mycovirus HetPV15-pa1 to a pre-infected host was elevated from zero to 50% by the presence of HetPV13-an1, and a double infection of these viruses in the donor resulted in an overall transmission rate of 90% to a partitivirus-free recipient. On contrary, pre-existing virus infections of two closely related strains of HetPV11 hindered each other's transmission, but had unexpectedly dissimilar effects on the transmission of more distantly related viruses. The co-infection of HetPV13-an1 and HetPV15-pa1 significantly reduced host growth, whereas double infections including HetPV11 strains had variable effects. Moreover, the results showed that RdRp transcripts are generally more abundant than capsid protein (CP) transcripts and the four different virus strains express unique transcripts ratios of RdRp and CP. Taken together, the results show that the interplay between co-infecting viruses and their host is extremely complex and highly unpredictable.

## Introduction

Fungi are known to host a wide variety of mycoviruses (Vainio and Hantula, 2018). Unlike other viruses, fungal RNA viruses lack extracellular infective particles, and are transmitted only via intramycelial anastomosis contacts and sexual or asexual spores (Ghabrial and Suzuki, 2009; Son et al., 2015; Vainio et al., 2015b). These viruses replicate within their host's cytoplasm or mitochondria and usually cause no visible phenotypic changes, although both adverse and mutualistic effects have been described (Huang and Ghabrial, 1996; Lakshman et al., 1998; Preisig et al., 2000; Ahn and Lee, 2001; Márquez et al., 2007; Yu et al., 2010; Hyder et al., 2013; Xiao et al., 2014; Vainio et al., 2018b). Some mycoviruses are used as biocontrol agents as demonstrated by the highly successful control of the chestnut blight fungus, *Cryphonectria parasitica*, by hypoviruses in Europe (Milgroom and Cortesi, 2004).

*Heterobasidion annosum* s.lat. species complex includes some of the most devastating infectious agents of conifer forests in the Northern Hemisphere (Garbelotto and Gonthier, 2013). About 15–17% of *Heterobasidion* strains are infected by one or more viruses (Vainio et al., 2013; Kashif et al., 2015; Vainio and Hantula, 2016). The most common species is the taxonomically unclassified Heterobasidion RNA virus 6 (HetRV6) which accounts for 70% of double stranded RNA (dsRNA) infections in *Heterobasidion* isolates of European origin, but also viruses of the families *Partitiviridae* and *Narnaviridae* are known to inhabit *Heterobasidion* mycelia (Ihrmark, 2001; Vainio et al., 2011b, 2012, 2015a). Both partitiviruses and HetRV6 may transmit across vegetatively incompatible or distantly related host isolates of *Heterobasidion* in laboratory and natural forest environments (Ihrmark, 2001; Vainio et al., 2010, 2011a,b, 2013, 2015b). In addition, uncharacterized dsRNA elements have been shown to be present in both basidiospores and conidia (Ihrmark et al., 2002, 2004), but only HetRV6 has been identified in basidiospores (Vainio et al., 2015b).

Most of the mycovirus species in *Heterobasidion* spp. belong to the family *Partitiviridae* with more than 20 species observed in these fungi. Partitiviruses have genomes composed of two segments of dsRNA packed in separate protein capsids and encoding for a putative RNA-dependent RNA polymerase (RdRp) and a capsid protein (CP) (Ihrmark, 2001; Vainio et al., 2010, 2011a,b, 2013; Kashif et al., 2015). The ratio of the RdRp and CP segments and their transcripts in infected mycelia usually deviates from 1:1, and is virus species specific but responds to environmental conditions (Jurvansuu et al., 2014).

Partitiviruses of *Heterobasidion* spp. are mostly cryptic or have only slight effects on their hosts (Vainio et al., 2010, 2012), but Heterobasidion partitivirus 13 strain an1 (HetPV13-an1) originally observed in *H. annosum* causes serious growth debilitation in both *H. annosum* and *H. parviporum* (Vainio et al., 2018b). Also in other fungi, partitiviruses have been shown to cause variable phenotypical changes or hypovirulence (Magae and Sunagawa, 2010; Bhatti et al., 2011; Xiao et al., 2014; Zheng et al., 2014; Zhong et al., 2014; Sasaki et al., 2016).

Multiple virus infections are common among *Heterobasidion* strains (Vainio et al., 2012, 2013, 2015a,b; Kashif et al., 2015; Hyder et al., 2018) as well as other fungi such as *Gremmeniella abietina* (Tuomivirta and Hantula, 2005; Botella et al., 2013), *Rhizoctonia solani* (Lakshman et al., 1998), *Helminthosporium victoriae* (Ghabrial et al., 2013), *Cryphonectria parasitica* (Peever et al., 1997), and *Sclerotinia sclerotiorum* (Marzano et al., 2015). The interactions between co-infecting viruses and their hosts may have a major effect on the ecology of both fungi and their viruses. Such interactions have recently been studied also among few other fungal viruses (Hillman et al., 2018). As one extreme, Zhang et al. (2016) showed mutualistic interactions in two virus strains, where the capsidless positive-sense ssRNA virus yado-kari virus 1 (YkV1) takes over the capsid protein of a dsRNA virus yado-nushi virus 1 (YnV1), and appears thereafter to replicate like a dsRNA virus. Also both a megabirnavirus (Sasaki et al., 2016) and a chrysovirus (Wang et al., 2014) have been shown to confer hypovirulence on their hosts when in coinfection with a partitivirus. In addition, the presence of Rosellinia necatrix mycoreovirus 3 (RnMyRV3) in recipient mycelia restricts horizontal transmission of Rosellinia necatrix partitivirus 1 (RnPV1) and Rosellinia necatrix megabirnavirus 1 (RnMBV1) (Yaegashi et al., 2011).

We tested whether the presence of other viruses in the donor or recipient affects the probability of viral transmission, and whether this effect depends on the taxonomic similarity of the co-infecting viruses in *Heterobasidion annosum*. We also determined the effects of viral co-infections on the host's growth rate and on the ratio of viral RdRp and CP transcripts in the mycelium, and compared the results to those measured from single infections.

## Materials and Methods

### Virus Strains

In order to test the effect of similarity of co-infecting viruses on transmission efficacy, phenotypic outcome of infection, and viral transcript levels, we selected two very closely related Heterobasidion partitivirus 11 strains, HetPV11-au1 and HetPV11-pa1 with 99% RdRp identity at the amino acid level (aa), and two moderately related partitiviruses, HetRV13-an1 and Heterobasidion partitivirus 15 strain pa1 (HetPV15-pa1) sharing 68% (aa) identity, being only distantly related (with 31% aa identity) to the other two viruses (Supplementary Table 1). HetPV11-au1 was originally hosted by *H. australe* strain 06111 of the *H. insulare* complex and HetPV11-pa1 by *H. parviporum* 06101 of the *H. annosum* complex. The RdRp encoding genome segments (Genbank accessions HQ541328 and HQ541329) of both strains have been characterized previously (Vainio et al., 2011a). In this study, we characterized the complete sequences of the CP encoding genome segments of both HetPV11 strains in order to determine their identity and screen their transmission between fungal strains.

The fungal strains *H. australe* and *H. parviporum* hosting HetPV11-au1 and HetPV11-pa1 were cultivated on MOS agar plates. The dsRNA was isolated by cellulose affinity chromatography and agarose gel electrophoresis followed by purification (Jurvansuu et al., 2014). Complementary DNA was made by PCR amplification using tagged random hexamer primers (Márquez et al., 2007) and thereafter cloned as previously (Vainio et al., 2011a). Sequence ends were determined by adapter ligation and PCR amplification with specific primers (Lambden et al., 1992). The sequences were compared to other viruses in GenBank using MAFFT alignment and NCBI BlastP.

### Preparation of Single Virus Infected Donor and Recipient Fungal Strains

Two heterokaryotic isolates of *Heterobasidion annosum* (03021 and 94233) were used as viral hosts in this investigation (Table 1). No viruses have been observed in the original isolate 03021 whereas isolate 94233 was the native host of HetPV13-an1 and hosted also cryptic mitoviruses (Vainio et al., 2015a, 2018b). The 94233-32D strain used in this study had been cured of HetPV13-an1 by thermal treatment (Vainio et al., 2018b).

**Table 1:** Fungal isolates and alphapartitivirus strains and their relevance.

**Fungal isolate**	**Partitivirus infection**	**Relevant information**	**Origin**	**References**
*H. annosum* 94233	HetPV13-an1	Virus causes serious disease on the host	Poland 1994	Kashif et al., 2015; Vainio et al., 2018b
*H. annosum* 94233/32D	None	94233 cured of HetPV13-an1, normal growth rate	Poland	Vainio et al., 2018b
*H. parviporum* 95122	HetPV15-pa1	Virus shares 68% RdRp aa identity with HetPV13-an1	Russia 1995	Kashif et al., 2015
*H. annosum* 03021	None	dsRNA free isolate	Finland 2003	Unpublished, Kari Korhonen, Luke culture collection
*H. parviporum* 06101	HetPV11-pa1	Cryptic virus sharing 97% RdRp nt identity with HetRV11-au1	Bhutan 2006	Vainio et al., 2011a
*H. australe* 06111	HetPV11-au1	Cryptic virus sharing 97% RdRp nt identity with HetRV11-pa1	Bhutan 2006	Vainio et al., 2011a

**Figure 1:**
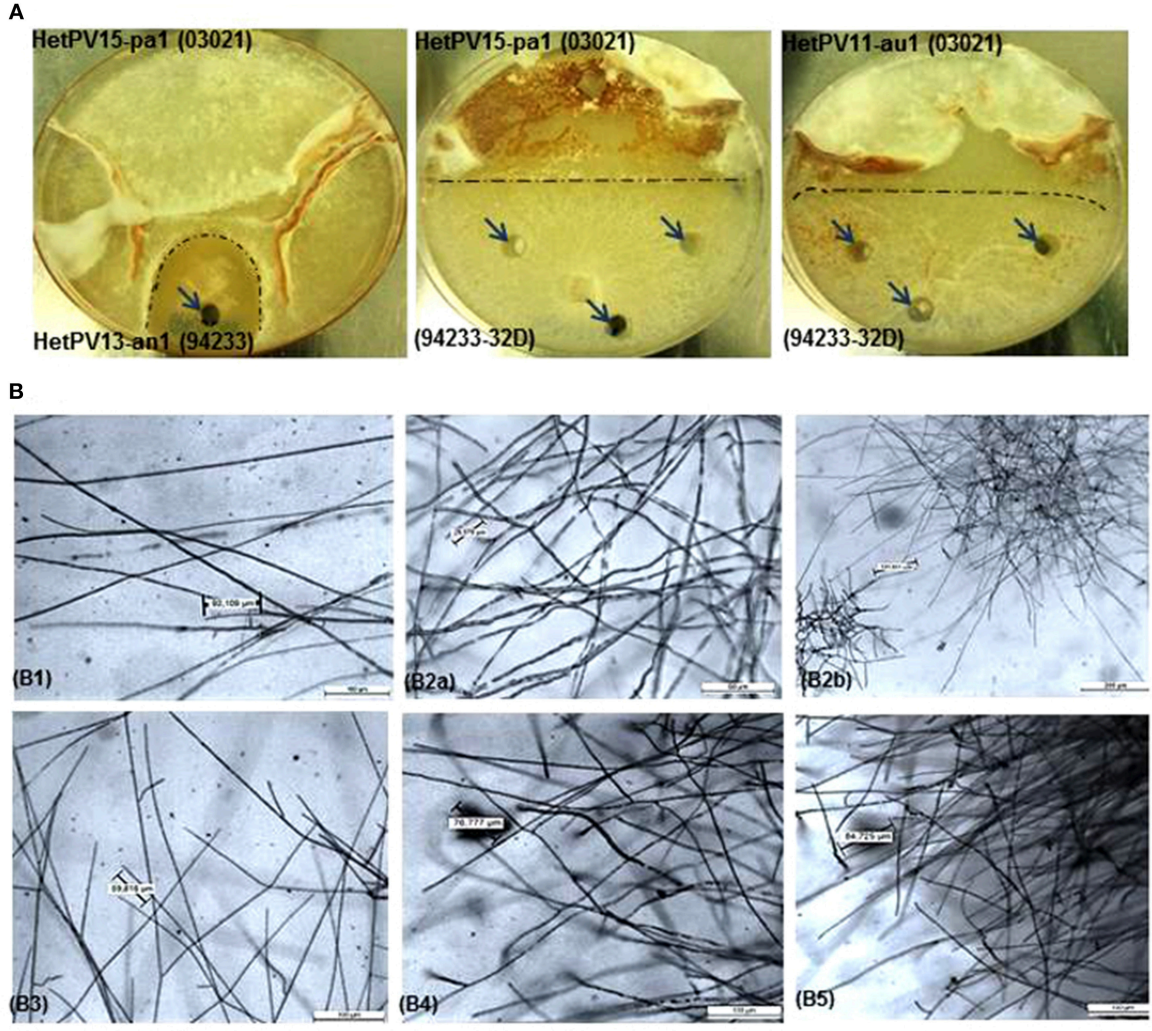
Hyphal phenotype and morphology of *Heterobasidion* strains with or without partitivirus infection, **(A)** Phenotype of dual cultures showing the demarcation area (dark dashed lines) between donor and recipient isolates and sampling spots shown by arrows, **(B)** morphology of fungal cultures under light microscope in different magnifications with eyepiece 10X/20M before and after infection with alphapartitiviruses, (B1) virus-cured 94233 (94233/32D(-)) shows normal less branched growth of hyphae (Objective; 10X/0.40), (B2a) native *H. annosum* 94233 infected with HetPV13-an1 (94233(HetPV13-an1)) displaying extensively branched hyphae (Objective; 20X/0.40) and (B2b) microscopic view of accumulated stunted hyphal growth in native 924233(HetPV13-an1) in lower magnification (5X/0.12). Host morphology (B3) transmission of HetPV13-an1 from the donor (03021) to recipient fungal host (94233) shows the same hyphal appearance (Objective; 10X/0.40) as shown in the native host (94233(HetPV13-an1)), (B4) hyphal growth (Objective; 10X/0.40) in 94233 infected with HetPV15-pa1 (94233(HetPV15)) after having lost HetPV13-an1 during dual culture and (B5) 94233 co-infected by HetPV13-an1 and HetPV15-pa1 exhibiting restricted and debilitated hyphal growth [(Objective; 10X/0.40)] as shown above in B2.

Virus transmission was examined using *H. annosum* 03021 as a virus donor and *H. annosum* 94233/32D as a recipient. However, the original 94233 isolated from the nature was used as the HetPV13-an1 hosting recipient. Prior to the experiments, each of the four virus strains were transmitted individually to the donor strain 03021 (not known to host viruses) (Vainio et al., 2011a,b) and to the recipient strain 94233-32D to obtain single infections of all partitiviruses in both strains. These transmissions were done using original or non-native hosts of these viruses (Supplementary Table 2) using dual cultures on single malt extract agar (MEA) plates incubated at 20°C for 4–8 weeks until the formation of clear hyphal contacts and a demarcation zone indicating somatic incompatibility between the strains (Figure 1A) as described previously (Ihrmark et al., 2002; Vainio et al., 2013). After the incubation, subcultures were established from the recipient side of the plate and the presence of viruses was examined using RT-PCR and host identity as a recipient with a pairing test (see below). However, transfer of HetPV15-pa1 to the recipient was not successful, and therefore 94233 with single HetPV15-pa1 was obtained by screening single hyphal tips of coinfected (HetPV13an1 and HetPV15-pa1) isolates. Double infected strains of *H. annosum* 03021 were generated during the transmission experiments to single infected 94233-32D when HetPV15-infected strains of 03021 (donor) received viruses from 94233 infected by either HetPV11-pa1, HetPV11-au1, or HetPV13-an1 (Table 2).

**Table 2:** The transmission of virus strains were tested in donor strains for their possible reciprocal transmission.

	**Reciprocal-transmission Infecting virus in donor 94233 (Transmission efficiency)**			
Pre-existing virus in recipient 03021	HetPV13-an1	HetPV15-pa1	HetPV11-pa1	HetPV11-au1
HetPV13-an1		50% (3/6)	50% (3/6)	33% (2/6)
HetPV15-pa1	100% (6/6)		50% (3/6)	50%(3/6)
HetPV11-pa1	50% (3/6)	33% (2/6)		0% (0/6)
HetPV11-au1	50% (3/6)	60% (4/6)	0% (0/6)	

### Transmission Frequency Test

**Figure 2:**
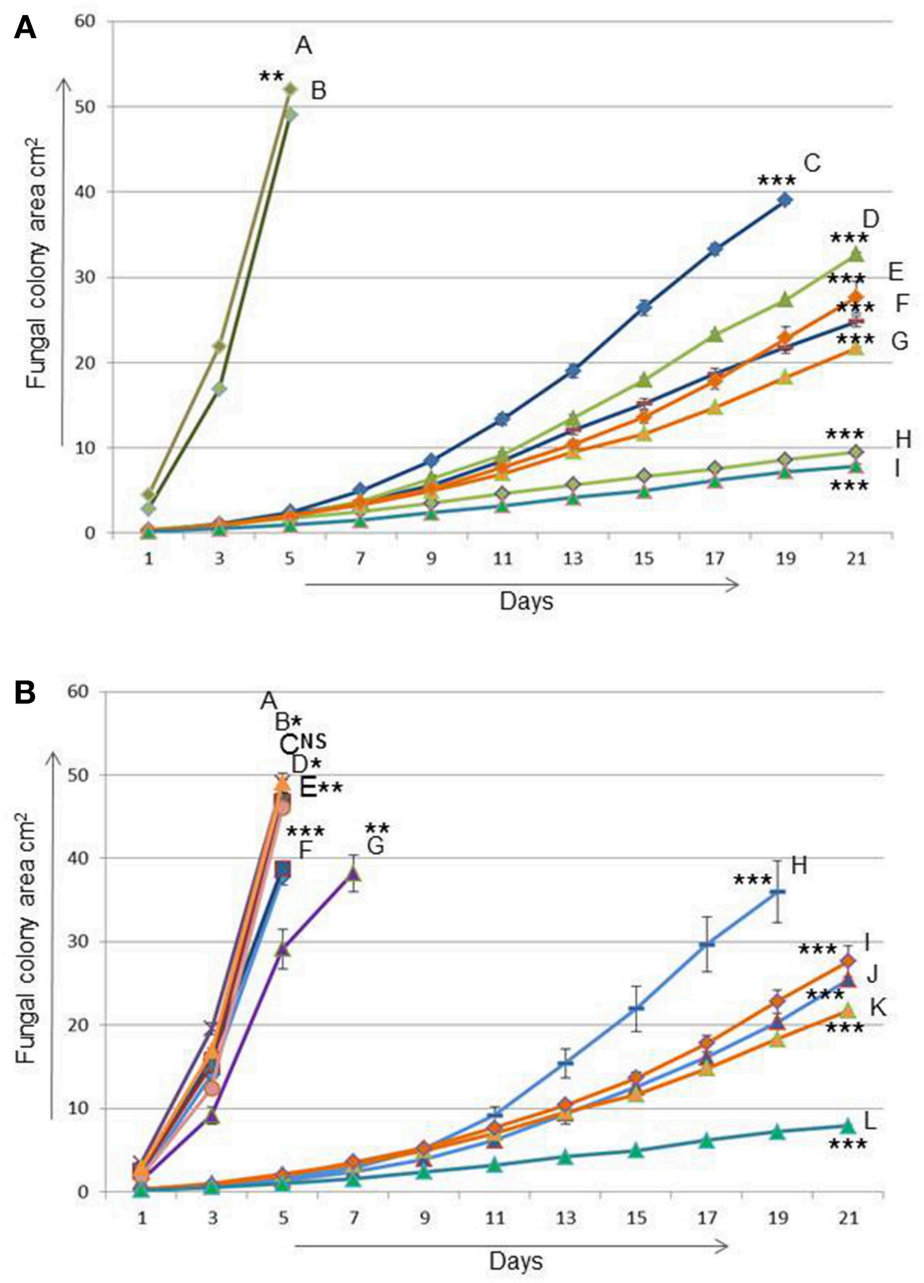
The effect of Heterobasidion partitiviruses in single and coinfection on the growth of their host after transmission from one fungal host (03021) to another (94233) **(A)** A = 03021 is naturally virus-free fungal strain (donor/*H. annosum*), B = 94233/32D is a fungal isolate 94233 (recipient/*H. annosum*) cured of virus HetPV13-an1, C and F = 15pa1 (8 and 6) are two separate fungal isolates (94233) infected by HetPV15-pa1, D and H = 15pa1+13an1 (8 and 6) are two independent isolates (94233) coinfected by HetPV15pa1 and HetPV13-an1, G and E = 13an1 (2 and 15) are two separate isolates (94233) where HetPV13-an1 was re-introduced to its native host and I = HetPV13-an1 infection in its native host (94233) **(B)** A = 94233/32D, B and C = 11pa1(1 and 9) isolates are infected by HetPV11-pa1, D and F = 11au1(1 and 7) by HetPV11-au1, E and G = 11au1+13an1(14 and 13) by coinfection of HetPV11-au1 and HetPV13-an1, H and J = 11pa1+13an1(3 and 5) by coinfection of HetPV11-pa1 and HetPV13-an1, I and K = 13an1(15 and 2) by HetPV13-an1 and L = HetPV13-an1 infection in its native host as mentioned above. 94233/32D and 03021 are fungal strains used as recipient and donor in transmission experiments, respectively. The error bars show standard errors of the means of twelve independent biological replicates. The significance of the differences between the mean growth per day of the virus-free/cured and virus infected fungal isolates was calculated with *t*-test and statistically significant differences are shown by asterisks: *P > 0.05, **P < 0.0001, and ***P < 0.00001, NS = Non-significant.

In order to test the effects of single viruses hosted by the recipient on the transmission of other viruses we made 20 dual cultures (Vainio et al., 2013) between each of the donors with one of the recipients with one or no partitiviruses on a single MEA plate (Figure 2A) resulting in a total of 320 transmission trials. After the incubation, subcultures were established from the recipient side of the plate and the presence of viruses was examined using RT-PCR (see below). In addition, six randomly selected plates from each donor-recipient pair were additionally sampled from the donor side in order to test whether reciprocal virus transmission had occurred.

Similarly, the effect of viruses to each other's transmission in a co-infection situation (two viruses in the same donor) was tested using HetPV15-pa1 in co-infection with either HetPV13-an1, HetPV11-au1, or HetPV11-pa1 in donor 03021, and using 94233-32D as a recipient. This experiment was conducted using 20 replicates.

In all experiments on MEA plates, mycelia was picked from recipient side of the culture using a sterilized Pasteur pipette with a diameter of 5 mm from 3 sampling points, unless the area occupied by the slowly growing recipient strain allowed sampling from only one spot. The three subcultures were then combined, cultured on modified orange serum (MOS) agar covered with a cellophane membrane and analyzed for the transmission of viruses using RT-PCR with both RdRp and CP primers (Supplementary Table 3). The significance of the difference between transmissions from hosts with different virus status was tested using Fisher's exact test.

### Fungal Strain Confirmation

#### Single Hyphal Tip Isolation

To get pure cultures of virus infected strains of *H. annosum* 03021 and 94233, three or four independently infected recipient subcultures from all obtained virus-host combinations were selected randomly for single hyphal tip isolations. The hyphal tips were collected under a binocular microscope with a modified Pasteur pipette using 1–3 day old cultures grown on 2% MEA as described by Vainio et al. (2010). The isolated hyphal tips were allowed to recover on MEA plates for 3–5 days before transfer to MOS agar plates covered by cellophane membranes. The mycelium was used to extract RNA for virus screening with RT-PCR and DNA for genetic fingerprinting to confirm the genotype of the recipient host. These purified cultures were then used for transmission, growth rate and RT-qPCR experiments.

#### Pairing Test and Genotyping (DNA Extraction and Microsatellite Markers)

The genotypes of the recipient strains were investigated using somatic compatibility tests (Stenlid, 1985) to verify their identity. In this experiment, the recipient subcultures were inoculated on a single plate with the original partitivirus-free recipient strain and the donor strain, and hyphal fusion was interpreted as a sign of genetic similarity. Additionally, the genotypes of all strains that had received viruses were analyzed using three different Random Amplified Microsatellites (RAMS) markers CT, CGA and CCA (Vainio and Hantula, 1999) which also clarified the cases of weak or unclear demarcation zones in somatic compatibility test.

### Growth Rate Measurements

The growth rate experiments were conducted using two strains (derivatives of 94233-32D) from each host/virus combination (either one or two viruses in the host) and partitivirus-free controls (94233-32D and 03021) as well as the original 94233 isolate with a natural infection of HetPV13-an1. The tested virus hosting isolates were obtained from the transmission experiments, and selected based on their apparent growth rates on single plates (as estimated by visual inspection) to cover the range of variation for each host/virus combination. The inoculum size was 0.5 cm in diameter and picked as circular agar plug one centimeter from the edge of the fresh mycelium and placed at the center of a 2% MEA plate. Twelve independent biological replicates were used for each isolate.

The fungal growth was recorded every second day after the mycelial growth was established in 3 days after inoculation. The growth was measured for 7–25 days depending on the growth rates of isolates. The area of fungal growth was measured by a digital planimeter (Planix 10S, Tamaya) and statistical difference was tested using *t*-test in Microsoft Excel 2010 (Supplementary Table 4).

#### Total RNA Isolation and cDNA Synthesis

Total RNA was extracted from fungal mycelia after one (controls or normally growing virus-infected fungal isolates) or 2 weeks (slowly growing single or co-infected fungal isolates) growth on MOS agar plates (Jurvansuu et al., 2014). In brief, fungal mycelia were collected and homogenized by 1–2 mm quartz sand in TRI Reagent (Molecular Research Center Inc., USA) in the Fast-Prep FP120 homogeniser (JT Baker, Holland). Total RNA was then isolated from the fungal sample by TRI Reagent according to manufacturer's recommendations. RNA pellet was eluted into DEPC treated water (G. Biosciences, USA) and the concentration and purity of the isolated RNA were analyzed by NanoVue (GE healthcare, USA).

Complementary DNA (cDNA) was made from 2 μg of DNase I treated total RNA using RevertAid First Strand cDNA Synthesis Kit (Thermo Scientific, USA) and random hexamer primers (Thermo Scientific, USA) as recommended by the manufacturer.

##### RT-qPCR Quantification for Virus Transcripts (RdRp and CP)

Half-sample volume of water was added to the cDNAs before using them in quantitative-PCR (qPCR). EvaGreen® dye (Solis BioDyne, Estonia) was used in qPCR on Rotor-GeneQ (Qiagen, USA) according to manufacturer's recommendations. PCR primers were used as follows: RNA polymerase II transcription factor (RNA pol2 TF) was used as a reference gene and specific primers based on RdRp and CP genomic segments of four virus strains including HetPV1-13an1, HetPV15-pa1, HetPV11-pa1, and HetPV11-au1 were used for virus transcripts (Supplementary Table 3). The CP and RdRp genes of each virus strain in the purified PCR product were cloned into TOPO-pCR2.1 vector using TOPO^®^ TA Cloning Kit (Invitrogen, USA) according to instructions by the manufacturer. The purified plasmids were then used in absolute quantification as shown by Jurvansuu et al. (2014). The normalization of the viral transcript levels was done using the host RNA pol2 TF as a reference gene (Raffaello and Asiegbu, 2013) and the absolute quantities of RNA transcripts or virus copy number were calculated using a standard curve.

## Results

### Sequence Properties of HetPV11 Capsid Protein

In order to be able to monitor the transmission and transcript levels of the CP-encoding genome segment and to determine the similarity of the genomes of the two different HetPV11 strains, the CP encoding genome segments of both conspecific strains were characterized (Supplementary Figure 1). The genomes of these viruses are composed of two linear dsRNA segments, the larger ones (2,033 and 2,029 bp for HetPV11-pa1 and HetPV11-au1, respectively) of which are coding for putative RdRps (Vainio et al., 2011a). The lengths of the smaller genome segments determined here were 1,818 bp (HetPV11-pa1) or 1,819 bp (HetPV11-au1) including 3′-terminal poly(A) tracts and encoded for a putative CP of 495 aa (nts 124-1611) with a GC content of ca. 52%. The sequences have been deposited to GenBank under the accession numbers MG948857 and MG948858. The CP encoding genome segments of HetPV11-au1 and HetPV11-pa1 share 97.2% nt and 99.8% aa sequence identity. Only one out of the total of 40 single nucleotide polymorphisms located in the CP ORF region results in an amino acid substitution, while the remaining ones are silent. As a comparison, the RdRp encoding genome segments of these virus strains share 97.7% nt sequence identity with 38 sequence polymorphisms in the ORF region causing five aa substitutions (99.2% aa sequence identity) (Vainio et al., 2011a).

The CPs of both HetPV11 strains shared 48% aa sequence identity with Heterobasidion partitivirus 1 (HetPV1-ab1; ADV15442.1), and significantly lower similarity (27% or less) with other viruses such as Diuris pendunculata cryptic virus (27% identity, AFY23215), Rhizoctonia fumigata partitivirus (26%, AJE25831), Amasya cherry disease-associated mycovirus (23%, YP_138536), Ceratobasidium partitivirus CP-b1 (25%, AOX47601), and Cherry chlorotic rusty spot associated partitivirus (23%, CAH03669). The RdRp aa identity between HetPV11 and HetPV1 was ~75% (Vainio et al., 2011a). These sequence similarities to other viruses were clearly below the species differentiation criteria for the family *Partitiviridae* (≤90% aa sequence identity in the RdRp and ≤ 80% aa sequence identity in the CP) (Vainio et al., 2018a), confirming that HetPV11 is a distinct species from its closest relative HetPV1 (note that HetPV11 was originally considered to be conspecific with HetPV1, and both viruses were previously named HetRV1; (Vainio et al., 2011a)). Based on ML phylogenetic analyses, the CP and RdRp sequences show similar overall phylogenetic affiliation with other viruses (Supplementary Figure 1 for CP, RdRp not shown).

### Transmission to Pre-Infected and Virus-Free Isolates

The transmission of four alphapartitivirus strains with different taxonomic relatedness in dual cultures resulted in the following findings: HetPV13-an1 had a transmission frequency of 25% to a partitivirus-free host, whereas HetPV11-au1 and HetPV11-pa1 transmitted in 45 and 65% of trials, respectively (Table 3). HetPV15-pa1 did not transmit at all to the partitivirus-free recipient even after additional 10 trials done after this experiment.

**Table 3:** The transmission efficiencies of alphapartitivirus strains from donor host (*H. annosum* 03021) to recipient fungal isolate (*H. annosum* 94233).

	**Virus strains in donor host 03021**			
**Pre-existing virus in recipient 94233**	**HetPV13-an1**	**HetPV15-pa1**	**HetPV11-pa1**	**HetPV11-au1**
Partitivirus free	25% (5/20)	0% (0/20)	65% (13/20)	45% (9/20)
HetPV13-an1		50% (10/20)	55% (11/20)	60% (12/20)
HetPV15-pa1	40% (8/20)		50% (10/20)	75%(15/20)
HetPV11-pa1	25% (5/20)	10% (2/20)		0% (0/20)
HetPV11-au1	70% (14/20)	60% (12/20)	15% (3/20)	

Transmission efficiency of HetPV15-pa1 was elevated from zero to 50% when the recipient was pre-infected with HetPV13-an1. Interestingly, RT-qPCR showed that HetPV13-an1 had disappeared from four recipients during or after the successful transmission of HetPV15-pa1. The reason for this remains unknown, but it should be noted that we confirmed the presence of HetPV13-an1 in the mycelium in inoculation source plates but not in plates where the transmission trial was conducted. The transmission efficacies of HetPV13-an1, HetPV11-pa1, and HetPV11-au1 to HetPV15-pa1 infected recipient were 40, 50, and 75%, respectively (Table 3).

HetPV11-au1 was not transmitted at all to a recipient pre-infected with HetPV11-pa1 and the transmission was poor also vice versa. The transmission efficacy of HetPV11-au1 to recipients pre-infected by HetPV13-an1 and HetPV15-pa1 were 60 and 75%, respectively. HetPV11-pa1 was transmitted to recipients with HetPV13-an1, HetPV15-pa1, and HetPV11-au1 with frequencies of 55, 50, and 15%, respectively (Table 3).

Statistical analysis on the differences between transmission rates to virus-free and pre-infected recipients indicated that six out of the 12 viral interactions were affected significantly by pre-infected viruses including transmission of HetPV13-an1 to pre-infected host with HetPV11-au1 (*P* = 0.005), HetPV15-pa1 to the hosts pre-infected with HetPV13-an1 (*P* < 0.001) or HetPV11-au1 (*P* < 0.001), HetPV11-au1 to the hosts with pre-existing viruses HetPV15-pa1 (*P* = 0.041) or HetPV11-pa1 (*P* < 0.001), and HetPV11-pa1 to the host containing HetPV11-au1 infection (*P* < 0.001).

As transmission in dual cultures allows transmission to both directions we also analyzed six isolates from each experiment from the donor side (reciprocal transmission; Table 2). Although such a small dataset does not allow meaningful qualitative analysis, the observations confirm that transmission of both HetPV11 strains are hampered by the presence of the other one in the recipient.

Taken together, Heterobasidion partitiviruses in recipient strains have highly variable effects on transmission of new viruses including both enhancement of transmission and mutual exclusion.

### Transmission From a Double Infected Isolate

Transmission of viruses from a double infected donor to a virus-free recipient was tested using three virus combinations. The transmission from a donor with both HetPV15-pa1 and HetPV13-an1 was very efficient with 75% frequency of both viruses and in addition 15% transmission frequency of only HetPV15-an1, thus resulting in a 90% overall transmission rate for HetPV15-pa1. Both changes were statistically significant (*P* < 0.001) compared to transmissions from the single infected strains. No transmission (0/20) was observed from donors with double infections by HetPV15-pa1 and HetPV11-pa1 (*P* < 0.001) or HetPV15-pa1, and HetPV11-au1 (*P* < 0.001) (Table 4).

**Table 4:** The transmission with co-infected strains in donor host (03021) to partitivirus-free recipient host (94233/32D) (nd = not determined).

**Recipient 94233-32D**	**Virus strains in donor host (03021)**		
Viruses transmitted	HetPV15-pa1 +HetPV13an1	HetPV15-pa1 +HetPV11-pa1	HetPV15-pa1 +HetPV11-au1
HetPV15-pa1	90% (18/20)	0% (0/20)	0% (0/20)
HetPV13-an1	75% (15/20)	nd	nd
HetPV11-pa1	nd	0% (0/20)	nd
HetPV11-au1	nd	nd	0% (0/20)

The transmission of both genomic segments RdRp and CP for all the replicates were screened by RT-PCR which consistently showed the transfer of both segments (and thus both particles) of each virus strain. Moreover, pairing tests and genotyping was conducted to confirm the recipient strain of all replicates of successful transmission.

This part of the research, as a whole, showed that HetPV15-pa1 had a significant positive effect on the transmission frequency of HetPV13-an1 and vice versa, whereas coinfecting HetPV15-pa1 prevented completely the transmission of HetPV11 strains.

### Growth Rates of Hosts With Single and Double Virus Infections

#### HetPV13-an1 and HetPV15-pa1 Caused Growth Debilitation

The presence of HetPV13-an1 with or without co-infecting viruses caused an unusual hyphal morphology characterized by dense, stunted, and copiously branched hyphae, whereas the occurrence of other viruses or virus combinations was associated with normal hyphal morphology (Figure 1B).

The viral effects on host's growth rate were measured using two independently infected *H. annosum* 94233 strains with 12 parallel measurements for each host/virus combination. *H. annosum* 94233-32D derivatives with single infection of HetPV13-an1 had 87 and 89% reduction in their growth rates (Figure 2A). Interestingly, the original isolate 94233 with naturally infected HetPV13-an1 had an even slower growth rate: 96% reduction compared to the cured isolate with no partitiviruses. All these differences were significant (Supplementary Table 4).

In the case of HetPV15-pa1 the two independently infected *H. annosum* 94233-32D isolates showed 88 and 80% reduced growth rates. In coinfection situation the two double infected (HetPV13-an1 and HetPV15-pa1) strains of 94233-32D had 84 and 95% reduction in their growth rates. The growth rates of these two strains differed significantly from each other and also from the controls and all other isolates (Figure 2A).

#### Variable Growth Effects by Conspecific Virus Strains on Their Fungal Host

Neither of the HetPV11 viruses affected the growth of *H. annosum* 94233-32D as single infections (Figure 2B). Also a double infection by HetPV11-au1 and HetPV13-an1 had no or very little effect on its host. However, the 94233-32D strains co-infected by HetPV11-pa1 and HetPV13-an1 showed significant growth debilitation up to 88% with a high range of variation, as half of the twelve individual subcultures displayed irregular growth patterns with slow and fast growing sectors, whereas six subcultures grew slowly but uniformly. In one of the parallel isolates the growth effects were almost as severe as in a single infection of HetPV13-an1 whereas the other one grew almost 7% faster albeit considerably slower than the isogenic control strain 94233-32D (Figure 2B). Statistical significance was found only for strains with clear differences in their growth rates (Supplementary Table 4).

Taken together the results of growth rate analyses, the presence of co-infecting viruses HetPV13-an1 and HetPV15-pa1 is causing a stable and significant negative effect on their host, whereas the presence of HetPV11 strains has variable phenotypic effects on their hosts when present in combination with HetPV13-an1, and no effects as single infections.

### Coinfection of Alphapartitiviruses Affects Transcript Levels of Viral RdRps and CPs

**Figure 3:**
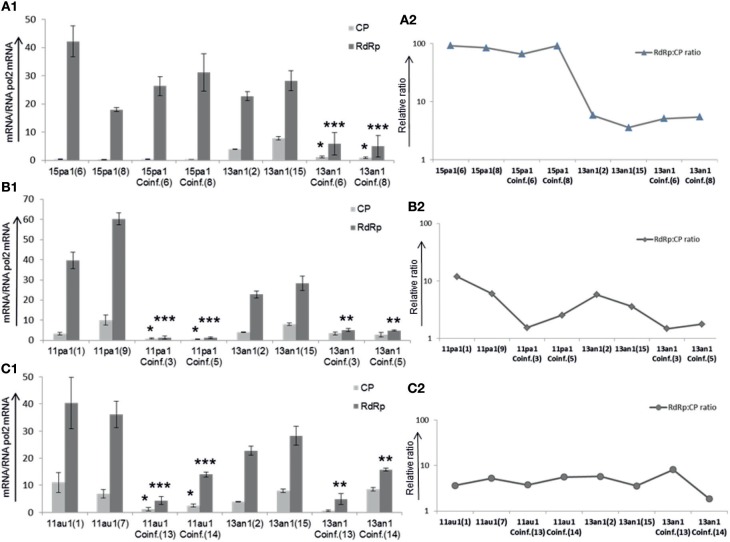
Heterobasidion partitiviruses in coinfection influence the amounts of viral CP and RdRp transcripts in recipient host (94233) **(A1)**. The quantification of viral transcripts was standardized to the amounts of *Heterobasidion* RNA pol2 mRNA. In comparison with HetPV15-pa1, the amounts of HetPV13-an1 transcripts were considerably reduced in coinfection **(A2)**. The relative ratio CP to RdRp of viral transcripts varied to lower or higher extent in each isolate of HetPV15, however the transcripts ratio for HetPV13-an1 remained the same after reduction in coinfection. 15pa1 (6 and 8) are two independent recipient fungal isolates (94233) infected by HetPV15-pa1, 15pa1/13an1Coinf (6 and 8) are two separate isolates coinfected by HetPV15-pa1 and HetPV13-an1 and 13an1 (2 and 15) are two isolates infected by solely HetPV13-an1 **(B1)**. over all amounts of HetPV11-pa1 (shown as 11pa1) transcripts were significantly reduced in coinfection **(B2)**. The relative CP to RdRp transcript ratios of HetPV11 isolates varied followed by considerable reduction whereas that of HetPV13-an1 remained the same after reduction in coinfection **(C1)**. In both isolates the amounts of viral transcripts were significantly affected in coinfection for HetPV11-au1 (shown as 11au1) including variable reduced amounts of HetPV13-an1 in each isolate of coinfection **(C2)**. The relative ratio CP to RdRp of viral transcripts varied to lower or higher extent in each isolate of HetPV11-au1 and HetPV13-an1. The linear scale is used due to variable expression of viral transcripts and log scale for their ratios. The error bars represent standard deviation of at least three separate experiments. The significance of the differences between the mean growth per day of the virus-free/cured and virus infected fungal isolates was calculated with *t*-test and statistically significant differences are shown by asterisks: *P < 0.05, **P < 0.001, and ***P < 0.0001.

The relative amounts of CP and RdRp viral sequences (copy number of transcripts and genome sequences; designated for simplicity as transcripts from now on) were studied using RT-qPCR for absolute quantification using the same fungal strains as in growth rate experiments. The amounts of transcripts of HetPV15-pa1 remain, on average, almost the same in single and coinfection with HetPV13-an1, although there was variation between the two independently created isolates (Figure 3A1). However, the quantity of transcripts of HetPV13-an1 was reduced significantly up to 4.8 and 4.6-fold for RdRp and CP in co-infection with HetPV15-pa1, respectively. Overall, the CP to RdRp transcript ratio of HetPV15-pa1 in two independent isolates was influenced on average by only 10.5% in coinfection. Also the overall change in the ratio between HetPV13-an1 CP and RdRp remained relatively stable (1:4 to 1:6) for single and coinfection (Figure 3A2), although the absolute quantities were leveled down (Figure 3A1).

Independent double infections by each of the highly similar strains of HetPV11 with HetPV13-an1 showed variable effects when compared with single infections of each of the three virus strains. Comparing the amounts of transcripts in coinfection to single infection, the quantity of viral transcripts for HetPV11-pa1 reduced drastically up to 9.6 and 40 times for CP and RdRp, respectively, whereas the CP and RdRp transcript levels of HetPV13-an1 were reduced only by 2 and 5 times, respectively (Figure 3B1), only RdRp change being significant. The ratio of CP to RdRp of HetPV11-pa1 changed from 1:5 to 1:13 in the two parallel strains in single viral infection to 1:1.6 and 1:2 in coinfection. The ratio for HetPV13-an1 reduced from 1:3.7 to 1:5 in the two parallel single infected isolates to 1:1.5 and 1:2 in coinfection (Figure 3B2).

Conversely, the overall amounts of viral transcripts of HetPV11-au1 were reduced only by 4 times (but significantly) in both viral segments in coinfection, whereas HetPV13-an1 showed 1.3- and 2.5-fold reduction in CP and RdRp transcript quantities in coinfection, only the RdRp change being significant (Figure 3C1). No clear direction of change was observed for CP to RdRp ratios of HetPV11-au1 and HetPV13-an1 in single and coinfections (Figure 3C2).

Overall, the quantity of viral transcripts of the virus strains was affected by coinfecting viruses except in the case of HetPV15-pa1.

## Discussion

In this study, we showed that both conspecific and relatively distantly related alphapartitiviruses affect each other's behavior transmission, phenotypic effects, and viral gene expression in a very complex way. It was expected that the two almost identical viruses (HetPV11-au1 and HetPV11-pa1) mutually hampered each other's transmission between two mycelia of *H. annosum* when present in the recipient (see e.g., Vainio et al., 2015b), but it was not expected that these two viruses would have very different effects on the transmission of more distantly related viruses: HetPV11-au1 in the recipient enhanced transmission of both HetPV13-an1 and HetPV15-pa1 considerably, whereas HetPV11-pa1 had no significant effect. However, and despite this, neither of the HetPV11 strains affected the transmission of HetPV15-pa1 when present in the same donor mycelium. Increase of transmission by other (co-infecting) viruses was most remarkably seen in HetPV15-pa1, which did not transmit at all without other viruses, but moved very efficiently when HetPV13-an1 co-infected the donor. These viruses have not been found to co-occur naturally, but in our experiments both of them acted as reciprocal helpers as the transmission rate of both viruses increased when they co-infected the donor. As the *Heterobasidion* strain 94233 used in this study was found to host mitoviruses only during the experiments reported here (Vainio et al., 2015a), we did not test their effects on the interactions studied here. Neither did we test the possible effects of host genotype on the virus transmission or phenotypic effects. It should, however, be noted that both mitovirus infection and the host strain may affect the interaction.

Virus transmissions occur frequently among *Heterobasidion* strains in laboratory and in nature, and even between related species (Ihrmark et al., 2002; Vainio et al., 2010, 2013, 2015b, 2017) but no quantitative data has been available on the mutual effects of different viruses transmissions before this study. Here we showed that in the case of HetPV15-pa1 the transmission of a virus occurred either infrequently or not at all between the donor and recipient, but was considerably enhanced by the presence of HetPV13-an1. In regard to HetPV15-pa1, it should also be noted that its transmission rates alone might be higher in nature than on artificial medium as shown previously by Brusini and Robin (2013) for the transmission rates of CHV1 of *C. parasitica*.

It has been shown that mycoviruses may occasionally transmit over fungal species borders (Yaegashi et al., 2013; Yaegashi and Kanematsu, 2016; Vainio et al., 2017; Arjona-Lopez et al., 2018). It has also been well-demonstrated that the transmission rates of fungal viruses depend on their host genome and genetic conservation of the host community (e.g., Biella et al., 2002; Brusini and Robin, 2013). Information from the interactions among other fungal viruses is more restricted, but it has been found that Mushroom bacilliform virus may require a helper-virus, LaFrance isometric virus, for its efficient transmission (Romaine and Schlagnhaufer, 1995). This study adds further evidence for this view by showing that lateral transmission of viruses is strongly affected by other viruses. However, the mechanism(s) of increased transmission rates by co-infecting Heterobasidion viruses remain unsolved, but it has previously been found that Sclerotinia sclerotiorum mycovirus 4 (SsMYRV4) changes the transcription and phenotype of the host fungus so that somatic incompatibility becomes leaky and enhances transmission of other viruses (Wu et al., 2017).

The phenotypic effects of mycovirus double infections on *H. annosum* were highly unexpected. HetPV13-an1 and HetPV15-pa1 caused considerable reduction in the growth rate of their hosts both alone and in coinfection. This is in accordance with previous results showing that these two viruses belong to the same clade among alphapartitiviruses (Kashif et al., 2015) and HetPV13-an1 causes a serious disease on both *H. annosum* and *H. parviporum*, and has a dramatic effect on their gene expression (Vainio et al., 2018b). Furthermore, the double infection by these viruses had a highly negative phenotypic effect (measured here as growth rate) although in some instances, genetically identical fungal strain-virus combinations with dissimilar infection histories (i.e., originating from different transmission trials) had somewhat deviating behavior. This was seen in e.g., the degree of growth rate reduction in strains infected independently by HetPV15-an1, and also between the independent strains with co-infection of HetPV13-an1 and HetPV15-pa1. We also observed considerably different degrees of variation in growth rates between parallel (technical) replicates of strains independently infected by HetPV15-pa1 (i.e., biological replicates). The history of infection expressed itself also as a difference in the growth rates of HetPV13-an1 infections in the original 94233 and 94233-32D strains. Such phenomena have not been reported previously, but these curious findings may point toward the importance of the development of virus-virus-host interactions during the very early infection, when the balance between the viruses and host develops.

Although the presence of HetPV13-an1 with other virus strains usually caused debilitation of its host's growth rate, the coinfection with HetPV11-au1 was an exception as its presence in the mycelium blocked the growth debilitation by HetPV13-an1. This was highly unexpected as the almost identical HetPV11-pa1 had no or a very limited effect. Recalling that the two conspecific HetPV11 strains had also different effects on the transmission of other viruses, they appeared to have a fundamental difference toward co-infecting viruses. It is not known how often highly similar viruses' effects on their hosts differ. In this respect it is, however, interesting that the phenotypic effects of HetPV13-an2 (*H. annosum* S45-8) with 97% nt RdRp/CP similarity to HetPV13-an1 (Kashif et al., 2015; Hyder et al., 2018) did not show similar negative effects on its native host.

The relative amounts of CP and RdRp transcripts were previously shown to vary considerably between different partitiviruses, although each virus had a similar RdRp/CP ratio in different hosts (Jurvansuu et al., 2014). In this study, HetPV13-an1 and HetPV15-pa1 expressed up to 6 and 102 times higher amounts of RdRp than CP transcripts in single infection, respectively, which agrees with the previous study on HetPV13-an1 (Vainio et al., 2015a). No previous information was available on HetPV15-pa1, but the high RdRp/CP ratio in all viruses studied here challenges the previous view that the theoretically expected higher need for CP transcripts (120 proteins in a functional particle compared to one RdRp needed to form a full virus) would usually be reflected in the quantity of transcripts within mycelia (Jurvansuu et al., 2014). It should be noted that the standard curves for qPCR were obtained using cloned cDNA instead of *in vitro* synthesized viral transcripts, which may introduce some bias when comparing the transcript levels between different genome segments due to possible template discrimination.

In the case of HetPV13-an1, the number of viral transcripts was reduced considerably in mycelia infected by two viruses (instead of one), whereas HetPV15-pa1 had similar amounts of viral transcripts in single and coinfection (with HetPV13-an1) situations. The RdRp/CP ratio, however, remained very similar for both HetPV13-an1 and HetPV15-pa1 as in single infection. Very interestingly, Wu et al. (2010) showed that the replication of Botrytis cinerea mitovirus 1 (BcMV1) is suppressed by another associated RNA virus (BcMV1-S), although it did not influence the debilitation effects on *B. cinerea* caused by BcMV1. Neither did the co-presence of HetPV13-an1 or HetPV15-pa1 affect each other's negative phenotypic effects on the host.

In conclusion, the interactions between partitiviruses of *Heterobasidion* spp. are complicated. Furthermore, especially the effects of HetPV13-an1 and HetPV15-pa1 on each other's transmission frequency and host phenotype makes them highly promising in terms of biocontrol against *Heterobasidion* spp. due to their simultaneous high transmission frequency and negative phenotypic effects.

## Data Availability

The datasets generated for this study can be found in NCBI Genbank, MG948857, and MG948858.

## Author Contributions

MK, EV, and JH conceived and designed the experiments. MK and JJ performed the experiments. MK, JJ, EV, and JH analyzed the data and wrote the paper.

### Conflict of Interest Statement

MK, EV, and JH are employed by the Natural Resources Institute Finland which has a pending patent application on the combined use of two mycoviruses for biological control based on this work. The remaining author declares that the research was conducted in the absence of any commercial or financial relationships that could be construed as a potential conflict of interest.
