# Supplementary material for: Alphapartitiviruses of Heterobasidion Wood Decay Fungi Affect Each Other's Transmission and Host Growth
Source: Front Cell Infect Microbiol. 2019 Mar 26;9:64. doi: 10.3389/fcimb.2019.00064 (PMC6443826; doi:10.3389/fcimb.2019.00064)
Supplement: Supplementary file 1 [file Data_Sheet_1.PDF]

*Supplementary Material*

# Alphapartitiviruses of Heterobasidion wood decay fungi affect each other's transmission and host growth

**Muhammad Kashif***, **Jaana Jurvansuu, Eeva J Vainio, Jarkko Hantula**

*** Correspondence:** Corresponding Author: muhammad.kashif@luke.fi

## 1 Supplementary Data

Supplementary Material should be uploaded separately on submission. Please include any supplementary data, figures and/or tables. All supplementary files are deposited to FigShare for permanent storage and receive a DOI.

Supplementary material is not typeset so please ensure that all information is clearly presented, the appropriate caption is included in the file and not in the manuscript, and that the style conforms to the rest of the article.

## 2 Supplementary Figures and Tables

For more information on Supplementary Material and for details on the different file types accepted, please see here. Figures, tables, and images will be published under a Creative Commons CC-BY licence and permission must be obtained for use of copyrighted material from other sources (including re-published/adapted/modified/partial figures and images from the internet). It is the responsibility of the authors to acquire the licenses, to follow any citation instructions requested by third-party rights holders, and cover any supplementary charges.

### 2.1 Supplementary Figures

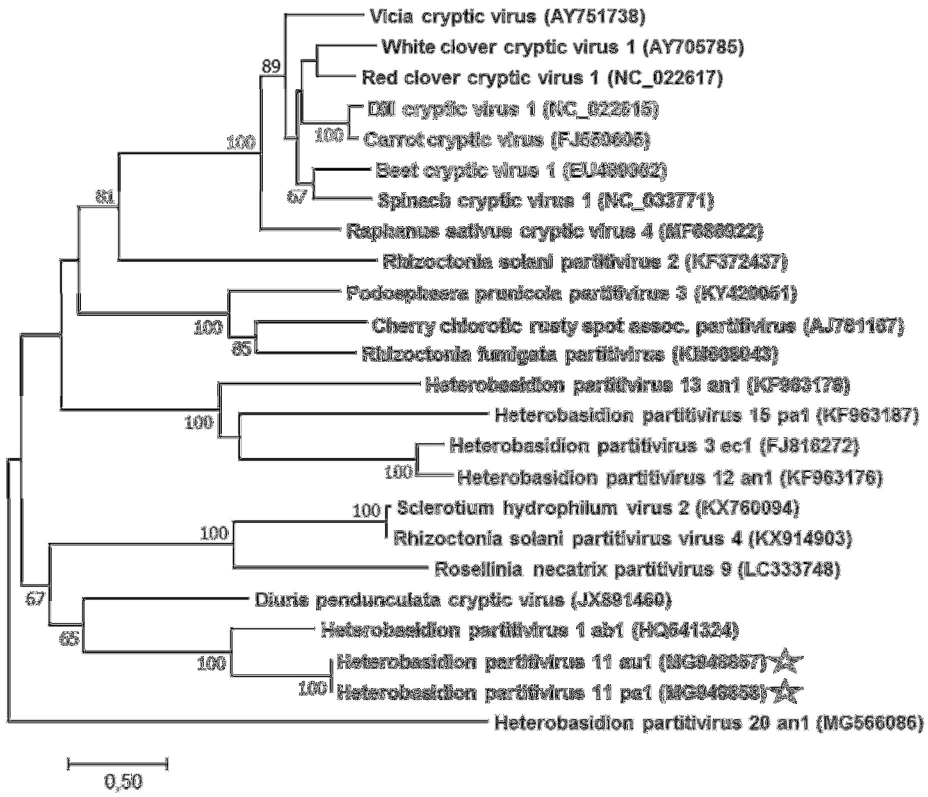


**Supplementary Figure 1.** Phylogenetic topology of putative protein sequences of selected partitiviruses using the maximum-likelihood method based on the Le Gascuel 2008 model (LG+G+I). Bootstrap support based on 100 repetitions is shown next to the branches when >50%. The tree is drawn to scale, with branch lengths measured in the number of substitutions per site. The aa sequence alignments were constructed by MAFFT, and evolutionary analyses were conducted in MEGA7 (http://www.mega software .net/). Analysis of CP aa sequences, including selected classified partitivirus species, all complete partitivirus CP sequences from *Heterobasidion* spp., and published (NCBI accession number) partitivirus CP sequences sharing over 50% based on BlastP identity with HetPV11 strains.

**Supplementary Table 1.** Sequence identity per cent matrix of Heterobasidion partitivirus coat protein (light grey) and RNA-dependent RNA polymerases (dark gray). In each cell, the upper value indicates nucleotide level similarity percentage and the corresponding amino acid level similarities are shown below. Matrix was created based on MAFFT alignment (www.ebi.ac.uk).

| Virus strains | | HetPV15-pa1 | HetPV13-an1 | HetPV11-pa1 | HetPV11-au1 |
|---|---|---|---|---|---|
| | | | CP (%) | | |
| HetPV15-pa1 | | | 44.0<br>28.9 | 35.4<br>18.3 | 35.1<br>18.3 |
| HetPV13-an1 | RdRp (%) | 65.5<br>67.6 | | 38.8<br>21.2 | 39.1<br>21.4 |
| HetPV11-pa1 | | 44.8<br>31.2 | 40.9<br>30.7 | | 97.2<br>99.8 |
| HetPV11-au1 | | 44.8<br>31.3 | 41.1<br>30.9 | 97.7<br>99.2 | |

**Supplementary Table 2.** The preparation of donor 03021 and recipient 94233 strains of *H. annosum* for the transmission of four virus strains.

| New host | Virus(es) | Donor host | Reference |
|---|---|---|---|
| 94233/32D | HetPV11-pa1 (06101) | *H. abietinum* 04070a | Vainio et al., 2011a |
| | HetPV11-au1 (06111) | *H. annosum* Ha_JH, | Vainio et al., 2011a |
| | HetPV13-an1 (94233) | *H. annosum* 94233 | Vainio et al., 2018 |
| | HetPV15-pa1 (95122) | *H. annosum* 94233 | Kashif et al., 2015<br>Vainio et al., 2018 |
| 03021 | HetPV11-pa1 (06101) | *H. parviporum* 06101 | Vainio et al., 2011a |
| | HetPV11-au1 (06111) | *H. australe* 06111 | |
| | HetPV13-an1 (94233) | *H. annosum* 94233 | Vainio et al., 2015a |
| | HetPV15-pa1 (95122) | *H. parviporum* 95122 | Kashif et al., 2015 |

**Supplementary Table 3.** The virus(es) genome based RdRp and CP primers used for the screening virus strains for checking transmission rates and qPCR quantification of transcripts (copy number). All the primers were designed and used in this study except the primer pairs HV3ConF1/HV3ConRe1 and PV15midF/PV15midRe (Kashif et al., 2015). (Annl. Temp. = Annealing temperature)

| **Primer description** | **Sequence**<br>**5'——————————>3'** | **Annl. temp. ℃** | **amplicon size (bp)** | **virus(es) detected** |
|---|---|---|---|---|
| HV3ConF1<br>HV3ConRe1 | ATG TTY TTC TGG CCY TTN TTC<br>GCG ANG TGR TCG AAG TAG TA | 56 | 608 | HetPV13-an1 (RdRp) |
| PV15midF<br>PV15midRe | TCC GAA GCT GAG CTA CAA CA<br>GGC TTT TGA AGT GCG GTA GA | 55 | 546 | HetPV15-pa1 (RdRp) |
| BHT866F<br>BHT1174R | ACC ATC TGG GGT GTT TCT AA<br>GCG AAG AGT CGC TTA AAC AT | 55 | 416 | HetPV11 (RdRp) |
| 1au1RdRp146F<br>1au1RdRp146R | CCTGCTTAAGATGCTCGCT<br>AATCGTGAATCTCCTTGAGG | 55 | 146 | HetPV11-au1 (RdRp) |

| | | | | |
|---|---|---|---|---|
| 1pa1RdRpF95<br>1pa1RdRpR95 | GATCATCTCCGAAGCTCAAATC<br>TGAACGTTTCGTAACCCCAG | 55 | 95 | HetPV11-pa1 (RdRp) |
| PV13an1CP.F1<br>PV13an1CP.R | CGA CGC TAT TCC AGC AAC TT<br>TGG TTG AAC GAG TTG AGC AG | 55 | 524 | HetPV13-an1 (CP) |
| PV15pa1CP.F<br>PV15pa1CP.R | TCG CTA ACT GCT CCG TCT TT<br>GGC GAG ATG GGA ATA GTT GA | 55 | 560 | HetPV15-pa1 (CP) |
| 1au1CP.rcm.F1<br>1au1CP.scrR1 | TTC GTC GTC CAC ATT CTT CG<br>GTT GAA GGG CAG ATG GAA TC | 55 | 387 | HetPV11-au1 (CP) |
| 1pa1CP.rcmF2<br>1pa1CPscr.R1 | ACC CTA ACA ACG CTG AAC AC<br>GGA AGA GGA AGG AGA CCA GT | 55 | 121 | HetPV11-pa1 (CP) |

**Supplementary Table 4 AB.** Statistical analysis by *t*-Test (two sample assuming unequal variances) in Excel 2010 to analyse the significance of variations in growth/day among 12 independent isolates (subcultures) for each sample for the growth rate. P-value; $H_0:\mu_1=\mu_2$ and $H_1:\mu_1\neq\mu_2$. $\alpha = 0.05$ (Single and coinfection in two groups A and B correspond to A and B in Figure 5).

| Virus strains **A** | | 15pa1 (8) | 15pa1 (6) | 13an1 (2) | 13an1 (15) | 15pa1+ 13an1(8) | 15pa1+ 13an1(6) | 13an1. Original |
|---|---|---|---|---|---|---|---|---|
| Growth/day ($cm^2$) | | **2.05** | **1.18** | **1.03** | **1.31** | **1.56** | **0.45** | **0.38** |
| P-values in the cells of above diagonal (dark) A and in below diagonal (light) B group | | | | | | | | |
| 11pa(9) | 0.445 | <0.001 | <0.001 | <0.001 | <0.001 | <0.001 | <0.001 | 15pa1(8) |
| 11pa1+ 13an1(3) | <0.001 | <0.001 | <0.001 | 0.021 | 0.547 | <0.001 | <0.001 | 15pa1(6) |
| 11pa1+ 13an1(5) | <0.001 | <0.001 | 0.004 | <0.001 | <0.001 | <0.001 | <0.001 | 13an1(2) |
| 11au1(1) | <0.001 | <0.001 | <0.001 | <0.001 | 0.003 | <0.001 | <0.001 | 13an1(15) |
| 11au1(7) | <0.001 | <0.001 | <0.001 | <0.001 | 0.477 | <0.001 | <0.001 | 15pa1+ 13an1(8) |
| 11au1+ 13an1(13) | <0.001 | <0.001 | <0.001 | <0.001 | 0.007 | 0.004 | <0.001 | 15pa1+ 13an1(6 |
| 11au1+ 13an1(14) | 0.149 | 0.034 | <0.001 | <0.001 | <0.001 | <0.001 | <0.001 | |
| 13an1(2) | <0.001 | <0.001 | 0.005 | 0.452 | <0.001 | <0.001 | <0.001 | <0.001 |
| 13an1(15) | <0.001 | <0.001 | 0.036 | 0.055 | <0.001 | <0.001 | <0.001 | <0.001 |
| 13an1. Org | <0.001 | <0.001 | <0.001 | 0.001 | <0.001 | <0.001 | <0.001 | <0.001 |
| Virus strains **B** | 11pa1 (1) | 11pa(9) | 11pa1+ 13an1(3) | 11pa1+ 13an1(5) | 11au1(1) | 11au1 (7) | 11au1+ 13an1(13) | 11au1+ 13an1(14) |
| Growth/day ($cm^2$) | **9.36** | **9.21** | **1.90** | **1.21** | **7.56** | **7.76** | **5.46** | **9.80** |
